# Extraction of the Bacterial Extracellular Polysaccharide FucoPol by Membrane-Based Methods: Efficiency and Impact on Biopolymer Properties

**DOI:** 10.3390/polym14030390

**Published:** 2022-01-19

**Authors:** Sílvia Baptista, Cristiana A. V. Torres, Chantal Sevrin, Christian Grandfils, Maria A. M. Reis, Filomena Freitas

**Affiliations:** 1 Associate Laboratory i4HB-Institute for Health and Bioeconomy, School of Science and Technology, NOVA University Lisbon, 2819-516 Caparica, Portugal; c.torres@fct.unl.pt (C.A.V.T.); amr@fct.unl.pt (M.A.M.R.); 2 UCIBIO—Applied Molecular Biosciences Unit, Department of Chemistry, School of Science and Technology, NOVA University Lisbon, 2819-516 Caparica, Portugal; 3 73100 Lda., Edifício Arcis, Rua Ivone Silva, 6, 4° piso, 1050-124 Lisboa, Portugal; silvia.baptista@73100.pt; 4 Interfaculty Research Centre of Biomaterials (CEIB), University of Liège, B-4000 Liège, Belgium; csevrin@uliege.be (C.S.); c.grandfils@uliege.be (C.G.)

**Keywords:** exopolysaccharide, fucopol, extraction, diafiltration, ultrafiltration, emulsion, rheology

## Abstract

In this study, membrane-based methods were evaluated for the recovery of FucoPol, the fucose-rich exopolysaccharide (EPS) secreted by the bacterium *Enterobacter* A47, aiming at reducing the total water consumption and extraction time, while keeping a high product recovery, thus making the downstream procedure more sustainable and cost-effective. The optimized method involved ultrafiltration of the cell-free supernatant using a 30 kDa molecular weight cut-off (MWCO) membrane that allowed for a 37% reduction of the total water consumption and a 55% reduction of the extraction time, compared to the previously used method (diafiltration-ultrafiltration with a 100 kDa MWCO membrane). This change in the downstream procedure improved the product’s recovery (around 10% increase) and its purity, evidenced by the lower protein (8.2 wt%) and inorganic salts (4.0 wt%) contents of the samples (compared to 9.3 and 8.6 wt%, respectively, for the previously used method), without impacting FucoPol’s sugar and acyl groups composition, molecular mass distribution or thermal degradation profile. The biopolymer’s emulsion-forming and stabilizing capacity was also not affected (emulsification activity (EA) with olive oil, at a 2:3 ratio, of 98 ± 0% for all samples), while the rheological properties were improved (the zero-shear viscosity increased from 8.89 ± 0.62 Pa·s to 17.40 ± 0.04 Pa·s), which can be assigned to the higher purity degree of the extracted samples. These findings demonstrate a significant improvement in the downstream procedure raising FucoPol’s recovery, while reducing water consumption and operation time, key criteria in terms of process economic and environmental sustainability. Moreover, those changes improved the biopolymer’s rheological properties, known to significantly impact FucoPol’s utilization in cosmetic, pharmaceutical or food products.

## 1. Introduction

Extracellular polysaccharides (EPS) are carbohydrate biopolymers secreted by the cells of many microorganisms that are released to the surroundings of the cells, remaining only loosely attached to them [1]. Many microbial EPS (e.g., xanthan and gellan gums, hyaluronic acid [2], and pullulan [3]) are utilized in high-value applications such as pharmaceutical, food, and cosmetic products [2,4,5] due to their biodegradable, biocompatible, and usually nontoxic nature [6,7].

Given their extracellular nature, EPS recovery from the culture broth involves rather simple downstream procedures that usually consist of cell removal, followed by biopolymer precipitation from the cell-free supernatant by addition of a precipitating agent (e.g., methanol, ethanol, isopropanol, acetone) and drying [1,8,9]. Although solvent precipitation with ethanol or acetone is the prevalent technique for EPS extraction, it usually yields rather impure polymers with high salts and/or protein contents [10]. Furthermore, this technique is expensive and impacts the environment due to the large volumes of used solvents and their handling/disposal [11]. Additional steps can be included in the downstream procedure, such as, for example, dilution of the broth for viscosity reduction or applying a heat treatment to kill bacteria and inactivate enzymes that could degrade the biopolymer during the process [11,12,13]. However, for high-value applications, in which a higher purity degree is often a prerequisite, specific procedures must be used to reach high-purity products. Such procedures include, for example, reprecipitating the EPS from dilute aqueous solutions, deproteinization by chemical or enzymatic methods, and membrane processes, such as dialysis, ultrafiltration and diafiltration [11,14,15]. Pressure-driven membrane processes, like diafiltration and ultrafiltration, are able to achieve high separation yields, while having a low environmental impact [16]. Nevertheless, the EPS rheological properties often cause membrane fouling, which reduces flux performance and increases operation times and, consequently, the processes’ operational costs [17]. Process optimization should therefore at least maximize the following criteria: recovery, highest purity, polymer performances adopting simple, rapid and green procedures. [1,18,19,20].

FucoPol is a fucose-rich EPS secreted by the bacterium *Enterobacter* A47 (DSM 23139) [21,22,23]. It is composed of neutral sugars, namely, fucose (32–36 mol%), galactose (25–26 mol%) and glucose (28–34 mol%), and the acidic sugar glucuronic acid (9–10 mol%). It also contains acyl groups: acetate (3–5 wt%), pyruvate (13–14 wt%), and succinate (3 wt%) [24]. FucoPol’s molecular weight was reported to range between 1.7 × 10^6^ and 5.8 × 10^6^ Da [22,23]. The presence of glucuronic acid, together with pyruvate and succinate, confers the biopolymer an anionic nature that promotes its interaction with cations and charged macromolecules [24,25]. FucoPol has proven valuable properties that include its thickening [24], filmogenic [26,27,28] and gelling capacity [22], as well as the ability to form and stabilize emulsions [3,23,27,29]. FucoPol recovery from the culture broth involves dilution with water for viscosity reduction, centrifugation for cell removal, heat treatment for enzyme inactivation and, finally, diafiltration and ultrafiltration of the cell-free supernatant with a 100 kDa molecular weight cut-off (MWCO) membrane [28]. Given the high viscosity of FucoPol aqueous media [30], the broth is usually diluted by 1:2 to 1:10 (*v/v*) [11,13,21,31,32], thus generating very large volumes for processing. Moreover, during the downstream procedures, product losses occur which decrease the process efficiency.

This study focused on optimizing the downstream procedure for FucoPol recovery from *Enterobacter* A47 cultivation broth. Three methods were designed and tested, using membranes of two different MWCO, namely, 100 kDa, which had been utilized in previous studies [13,33], and 30 kDa. The performance of each method was evaluated in terms of operating time, water consumption, and polymer recovery. The impact of the different purification methods on FucoPol’s physical-chemical properties, as well as on the biopolymer’s rheological properties and emulsifying behavior, were also evaluated.

## 2. Materials and Methods

### 2.1. FucoPol Production

FucoPol was produced by cultivation of *Enterobacter* A47 (DSM 23139) in a 10 L bioreactor (BioStat B-plus, Sartorius, Germany) operated under a fed-batch mode, using glycerol (40 g/L) as the carbon source, as described by Torres et al. [21]. The cultivation broth was collected and utilized for the extraction and purification experiments.

### 2.2. FucoPol Extraction and Purification

**Figure 1:**
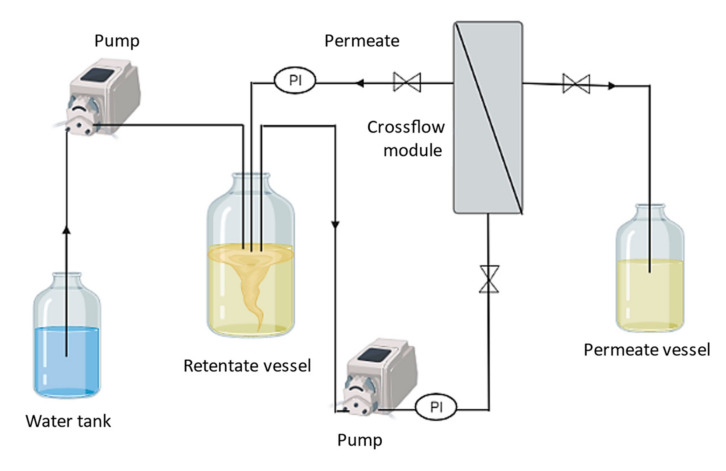
Schematic representation of the set-up used for the diafiltration/ultrafiltration experiments (PI—pressure gauge).

The culture broth (240 mL) was diluted with deionized water to a final volume of 2400 mL, and centrifuged (13,000× *g*, 45 min) for cell removal. The cell pellet was discarded, and the cell-free supernatant was subjected to a thermal treatment (70 °C, for 1 h) for enzyme inactivation [11]. The remaining cells and denatured proteins were removed by centrifugation (13,000× *g*, 45 min) and the resulting treated cell-free supernatant was used for the purification experiments that comprised three different methods, as described below. The set-up (Figure 1) included a crossflow module (Sartocon Slide Holder, Sartorius, Germany), using either a 100 kDa or a 30 kDa MWCO (Hydrosart, Sartorius, Germany), with a surface area of 0.1 m^2^. All experiments were performed at room temperature (~23 °C).

− Method 1 comprised a diafiltration step followed by an ultrafiltration step, as described by Meireles et al. [11] and was used as a reference method. Briefly, the procedure consisted in operating the module in a diafiltration mode, in which deionized water was continuously added to the supernatant vessel, in view to keep a volume constant in the retentate’s vessel (~2400 mL). When the retentate’s conductivity reached a value below 200 µS/cm, water addition to the vessel was suspended and the solution was concentrated to a volume of ~240 mL by operating the module in an ultrafiltration mode.
− Method 2 consisted in operating the crossflow module entirely in the ultrafiltration mode. The treated supernatant in the retentate vessel (2400 mL) was concentrated to a volume of ~240 mL. Afterwards, the retentate was diluted with deionized water to the initial solution’s volume and the ultrafiltration step was repeated. This procedure (ultrafiltration/dilution) was carried out until the retentate reached a conductivity below 200 µS/cm.
− Method 3 was identical to Method 1, exception made that the supernatant in the retentate vessel was first concentrated to 50% of its initial volume (~1200 mL). Then, during the diafiltration mode, deionized water was added to the retentate vessel to keep the volume at ~1200 mL, until the conductivity reached a value below 200 µS/cm. Finally, the ultrafiltration mode was implemented to concentrate the retentate to a final volume of ~240 mL.

At the end of the purification procedures, the concentrated retentate was freeze-dried (ScanVac CoolSafeTM, LaboGene, Lillerød, Denmark), at −110 °C for 48 h, for product quantification. The samples obtained with the 100 kDa and 30 kDa membranes were identified as F-i_100_ and F-i_30_, respectively (i = method 1, 2 or 3).

The inlet (P_in_, bar) and outlet (P_out_, bar) pressures, as well as the time (min) and the overall volume of water (L) required to reach a conductivity value below 200 µS/cm, were registered. The transmembrane pressure (TMP, bar) was determined using the following equation [34]:

(1) $$\mathrm{TMP}=\frac{\left(P_{\mathrm{in}}+P_{\mathrm{out}}\right)}{2}$$

### 2.3. FucoPol Characterization

#### 2.3.1. Chemical Composition

Freeze-dried FucoPol samples (5 mg) were dissolved in deionized water (5 mL) and hydrolyzed with 0.1 mL 99% trifluoroacetic acid (TFA) at 100 °C for 4 h, as described by Freitas et al. [27]. The monosaccharide contents in the hydrolysate were identified and quantified by liquid chromatography, using a Carbopac PA10 column (Thermo Scientific™ Dionex™, Sunnyvale, CA, USA), equipped with an amperometric detector. The analysis was performed at 30 °C with sodium hydroxide (NaOH 4 mM) as eluent, at a flow rate of 0.9 mL/min. The acyl substituents were quantified with an Aminex HPX-87H 300 × 7.8 mm column (Biorad, Hercules, CA, USA), coupled to an infrared (IR) detector, using sulfuric acid 0.01 N as eluent, at a flow rate of 0.6 mL/min and a temperature of 30 °C. All analyses were performed in triplicate.

#### 2.3.2. Elemental Analysis

Elemental analysis was performed in an Elemental Analyzer Thermo Finnigan-CE Instruments (Wigan, UK), model Flash EA 1112 CHNS, equipped with a gas chromatography (GC) and a thermal conductivity detector (TCD).

#### 2.3.3. Inorganic Salts Content

The total inorganic salts content of the samples was determined gravimetrically by incinerating 50 mg of dried polymer samples at 550 °C for 12 h.

#### 2.3.4. Molecular Mass Distribution

Molecular number (Mn), average molecular weights (Mw), and the polydispersity index (PDI = Mw/Mn) of FucoPol samples were obtained by size exclusion chromatography coupled with multiangle light scattering (SEC-MALS), as described by Torres et al. [35]. FucoPol solutions (2 mg/mL) were dissolved in 0.1 M Tris-HCl + 0.2 M NaCl, pH 8.09 buffer, which was also the SEC mobile phase. These solutions were warmed for 1 h at 80 °C under lateral agitation in a water bath. Dissolution of the polymer was continued for 24 h under a rocking roller at room temperature. The SEC columns (PL Aquagel-OH mixed 8 μm; 300 × 7.5 mm) protected by a guard column (Polymer Laboratory, Berkshire, UK; 50 × 7.5 mm, part no. 1149-1840) were equilibrated overnight before running the analysis at a flow rate of 1 mL/min at room temperature. Each analysis was conducted in duplicate. The purity and molecular mass distribution of the polysaccharide were monitored with MALS and RI detectors. These data were analyzed with Astra software (Santa Barbara, CA, USA) (V 4.73.04). A dn/dc of 0.190 mL/g was adopted to calculate the Mw.

#### 2.3.5. Fourier Transform Infrared (FT-IR) Spectroscopy

FT-IR spectroscopy with Diamond ATR (Attenuated Total Reflectance) was used to collect the spectra of the samples with a Perkin Elmer Spectrum Two (Perkin Elmer Inc., Waltham, MA, USA), equipped with a lithium tantalate (LiTaO_3_) detector with an SNR (signal to noise ratio) of 14.500:1. The resolution was 0.5 cm^−1^ and the number of scans was eight. The samples were placed in the absorbance chamber and corrected by applying the ATR-correction function of Perkin Elmer Spectrum (Waltham, MA, USA) software at the region of 4500–500 cm^−1^.

#### 2.3.6. Thermogravimetric Analysis (TGA)

TGA was performed using a Thermogravimetric Analyzer Labsys EVO (Setaram, France). The samples were placed in aluminum crucibles and heated from room temperature to 550 °C, with a heating rate of 10 °C/min, in air. The thermal degradation temperature (T_deg_, °C) corresponds to the temperature value obtained for the maximum decreasing peak of the sample mass.

### 2.4. Rheological Properties

The rheological properties of FucoPol aqueous solutions (1.0 wt%) were studied using a MCR 92 modular compact rheometer (Anton Paar, Madrid, Spain), equipped with a PP50/S parallel plate geometry (diameter 50 mm). The temperature was kept constant at 25 °C using a P-PTD 200/AIR Peltier plate (Anton Paar, Madrid, Spain. The flow curves were determined using a steady-state flow ramp in a shear rate range of 0.01 to 1000 s^−1^. The flow curves obtained were fitted to the equation based on Cross model [30] described as follows:

(2) $$\eta =\frac{\eta_{0}}{1+{\left(\tau \left.\dot{\gamma }\right)\right.}^{m}}$$

where *η* is the apparent viscosity (Pa·s), *η*_0_ (Pa·s) is the viscosity at zero shear rate, *τ* (s) is the relaxation time, and *m* is a dimensionless constant, related to the exponent of power-law (n) by *m* = 1 − *n* [24,27]. Frequency sweep tests were performed with frequency ranging from 0.01 to 100 rad/s with a constant strain of 0.5% that was well within the linear viscoelastic limit (LVE), which was evaluated through preliminary amplitude sweep tests. All tests were performed in triplicate.

### 2.5. Emulsion Forming and Stabilizing Capacity

The ability of the extracted FucoPol samples to stabilize emulsions was assessed by mixing 3 mL FucoPol (1.0 wt%) aqueous solution with 2 mL olive oil (purchased from a local market) to give a 2:3 (*v/v*) emulsion ratio. The mixtures were manually agitated for 40 s and left standing for 24 h, at room temperature. The emulsification index E24 (%) was determined using the following equation [27]:

(3) $$E_{24}=\frac{h_{e}}{h_{T}}\times 100$$

where h_e_ (mm) is the height of the emulsion layer and h_T_ (mm) is the overall height of the mixture. The rheological behavior of the different emulsions was evaluated as described above. All tests were performed in triplicate.

## 3. Results

### 3.1. Optimizing FucoPol Purification by Diafiltration and/or Ultrafiltration Procedures

At the end of the cultivation run, the culture broth had an apparent viscosity higher than 11 Pa·s (at a shear rate of 0.008 s^−1^). The broth was diluted with deionized water (1:10, *v/v*), for viscosity reduction, thus allowing for cell removal by centrifugation. The resultant cell-free supernatant was subjected to a thermal treatment to inactivate bacterial enzymes [11,23], and the treated cell-free supernatant was used for testing FucoPol recovery using membranes of two different MWCO, namely, 100 kDa and 30 kDa. Three methods were evaluated for each membrane (Table 1). The retentate’s conductivity was used to evaluate the purification progress: a value below 200 µS/cm was targeted as indicative of the elimination of most low Mw compounds from the sample (the treated cell-free supernatant had an initial conductivity value of 2800 ± 300 µS/cm). The extraction time was defined as the time required to reach the target for the retentate’s conductivity, followed by its concentration to the initial broth volume (~240 mL).

**Figure 2:**
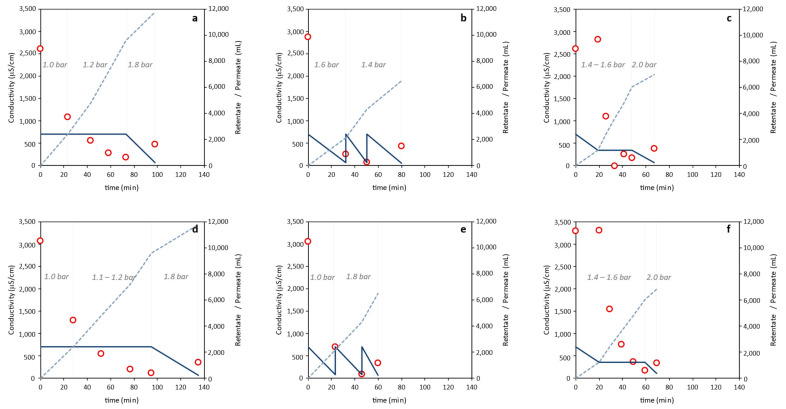
Retentate (full lines) and permeate (dashed lines) volumes, retentate conductivity (circles), and inlet pressure (italic), over time, for experiments F-1_100_ (**a**), F-1_30_ (**b**), F-2_100_ (**c**), F-2_30_ (**d**), F-3_100_ (**e**) and F-3_30_ (**f**).

**Figure 3:**
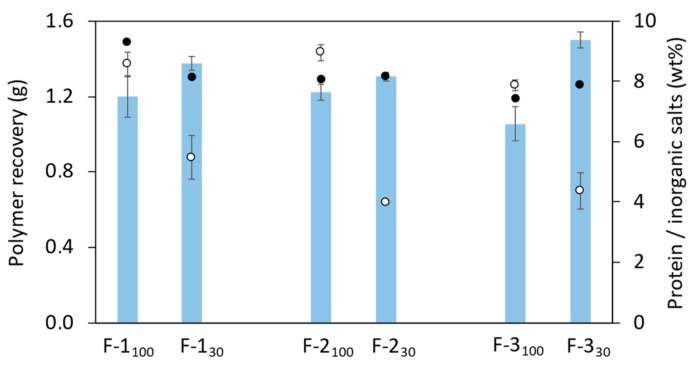
Polymer recovery (bars) and samples content in protein (closed circles) and inorganic salts (open circles) for the experiments performed with Methods 1, 2 and 3, with the 100 kDa and the 30 kDa membranes.

The results summarized in Table 1 and Figure 2a,b highlight that Method 1 (diafiltration-ultrafiltration) with either membrane involved the largest water consumption (9.6 L) and took the longest time (73–95 min) to reach the envisaged conductivity value (Table 1 and Figure 2a,b). Although the same TMP (0.60 ± 0.04 bar) was applied for both membranes, the extraction time with the 30 kDa membrane was higher due to the lower cut-off. The retentate’s conductivity decreased significantly during the initial 23–28 min, continuing to decrease afterwards until the target conductivity value was achieved (Figure 2a,b). The sample obtained after concentration of the retentate had a slightly higher conductivity as a concentration effect of the final ultrafiltration step. Compared to sample F-1_100_ (1.20 ± 0.11 g), a higher polymer recovery was noticed for sample F-1_30_ (1.38 ± 0.04 g). Interestingly, sample F-1_30_ had lower protein and inorganic salts contents, thus showing its higher purity (Figure 3).

A considerable reduction in water consumption (from 9.6 L to 4.3 and 6.0 L) was observed for Methods 2 (ultrafiltration) and 3 (ultrafiltration-diafiltration-concentration), together with a reduction of the overall extraction time (66–77 and 81–85 min, respectively) (Table 1, Figure 2). The sharpest drop in conductivity was observed for Method 2, where after the first ultrafiltration step (that took 30 min) it dropped down below 1000 µS/cm (Figure 2c,d). Similarly to Method 1, for Methods 2 and 3 the 30 kDa membrane gave rise to higher polymer recovery: 1.31 ± 0.05 and 1.50 ± 0.04 g, respectively, compared to the 100 kDa one (1.22 ± 0.04 and 1.06 ± 0.09 g, respectively) (Table 1). Interestingly, identical protein removal was achieved with both membranes in either method, as shown by the similar protein content in the samples (7.4–8.2 wt%). In contrast, salt removal from the supernatant was more efficient for the 30 kDa membrane (4.0–4.4 wt%, compared to 7.9–8.2 wt% for the 100 kDa membrane) (Figure 3).

### 3.2. Physical and Chemical Characterization of the Extracted FucoPol Samples

#### 3.2.1. Composition

The extracted FucoPol samples were characterized in terms of their sugar and acyl groups composition to assess the impact of the tested extraction and purification methods. As shown in Table 2, all samples had identical sugar composition, namely, fucose, galactose, glucose and glucuronic acid contents of 38–41 mol%, 24 mol%, 27–29 mol% and 6–7 mol%, respectively. This sugar monomer composition is similar to former data reported for glycerol-derived FucoPol [13,23,27,29,35,36] despite a slightly higher fucose content and a lower glucuronic acid content (Table 2). The higher fucose content renders the biopolymer more advantageous due to the biological activity reported for fucose-containing polysaccharides, namely, the reduction of allergic reactions, wound healing, antiaging [37,38], anti-inflammatory, and anticancer activities [39,40]. Regarding the acyl groups content, all samples had similar composition (5.1–5.4 wt% pyruvyl, 1.0–1.1 wt% succinyl and 4.4–5.4 wt% acetyl), in the ranges of previously reported values [21,23,27,35] (Table 2).

#### 3.2.2. Molecular Mass Distribution

The extracted samples had similar average Mw that ranged from 1.4 × 10^6^ Da to 2.0 × 10^6^ Da, with PDI values of 1.36–1.74 (Table 2). These values are comparable to those reported in previous studies for FucoPol (Mw = 1.7 × 10^6^ − 5.8 × 10^6^ Da and PDI = 1.3–1.9) [22,23,29,31], thus confirming that the applied extraction and purification methods had no significant impact on FucoPol’s molecular mass distribution. All samples contained polydisperse macromolecules, as expected for carbohydrate polymers, but given their low PDI values, their size distribution was rather narrow, indicating homogeneous FucoPol samples were produced by all tested procedures.

#### 3.2.3. FT-IR Spectroscopy

**Figure 4:**
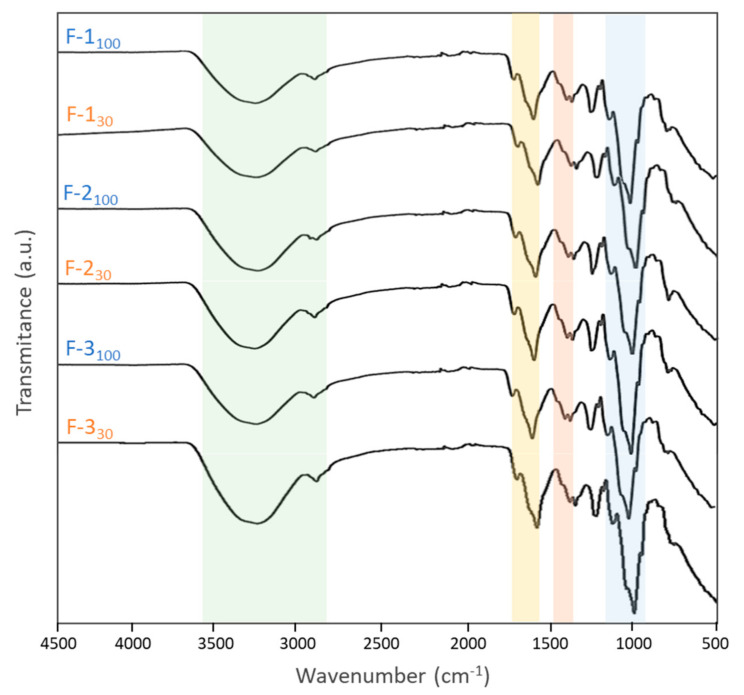
Comparative FT-IR spectra of the FucoPol samples extracted with Method 1 (F-1_100_; F-1_30_), Method 2 (F-2_100_; F-2_30_) and Method 3 (F-3_100_; F-3_30_).

The FT-IR spectra of the extracted FucoPol samples (Figure 4) are identical to those reported in the literature for FucoPol [13,23]. Common to all polysaccharides, two bands are observed around the 3280–2930 cm^−1^ region (Figure 4, green): the strong broadband appearing at 3282 cm^−1^ represents the O-H stretching of hydroxyls’ vibrations, and the weak signal at 2923 cm^−1^ is due to the C-H stretching peak of CH_2_ groups [13,41]. In addition, the IR absorption bands around 970-1145cm^−1^ (Figure 4, blue) are mainly due to C-C and C-O stretching in the pyranoid ring and C-O-C stretching of glycosidic bonds [42,43,44]. The bands observed at 1725 cm^−1^ (Figure 4, yellow) and at 1248 cm^−1^ may be attributed to the acyl substituents present in FucoPol’s structure, namely, the C=O stretching vibrations of carbonyls and the C-O-C vibrations, respectively [23,43]. The peak around 1603 cm^−1^ and the peaks in the region between 1400 and 1370 cm^−1^ (Figure 4, yellow and orange) may be assigned to the asymmetric and symmetric stretching of carboxylates from glucuronic acid [13,23,29]. The presence of bound water might be indicated by the bending vibration of O-H associated with a peak around 1603 cm^−1^ (Figure 4, yellow) [42,43,44].

#### 3.2.4. Thermogravimetric Analysis

**Figure 5:**
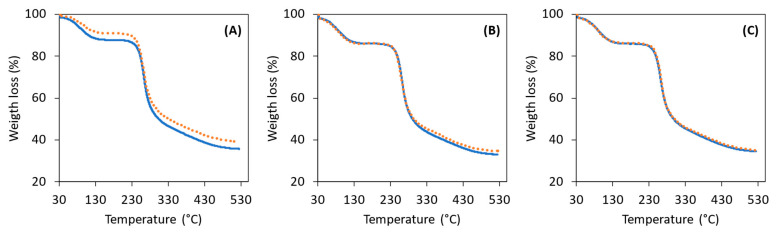
Thermal analysis curves for the FucoPol samples obtained by the different tested methods: (**A**) Method 1, (**B**) Method 2, (**C**) Method 3; F-i_100_, full blue line; F-i_30_, dotted orange line.

All extracted FucoPol samples displayed similar TGA curves, with two main degradation steps (Figure 5). The first degradation step, corresponding to weight losses of 8–12%, occurred between around 36 and 137 °C and is related to the elimination of water molecules physically entrapped or/and adsorbed to the polysaccharide through hydrogen bonding [45,46]. Samples F-1_100_ and F-1_30_ had the lowest weight loss values at this temperature range (10 and 8%, respectively), suggesting they had lower contents of adsorbed water compared to the samples obtained by Methods 2 and 3 that displayed the same weight loss (12%). The second and more significant weight loss, similar to all samples (41–43%), occurring between 203 and 344 °C, is the degradation of FucoPol’s saccharide chain, namely, the depolymerization and the dehydration of saccharide rings [47]. The T_deg_ of the samples was determined to be in the range 261–263 °C, thus showing no significant differences among the samples extracted by each of the tested methods. As the temperature further increases, there is the formation of polynuclear aromatic and graphitic carbon structures, resulting in the formation of a char that accounted for 33–40% of the samples’ mass. Interestingly, Methods 2 and 3 yielded FucoPol samples with lower char yield (33–35%) compared to those obtained with Method 1 (36–40%), which may be related to the samples’ lower content in inorganic salts (Figure 3).

### 3.3. Rheological Properties of the Extracted FucoPol Samples in Aqueous Medium

**Figure 6:**
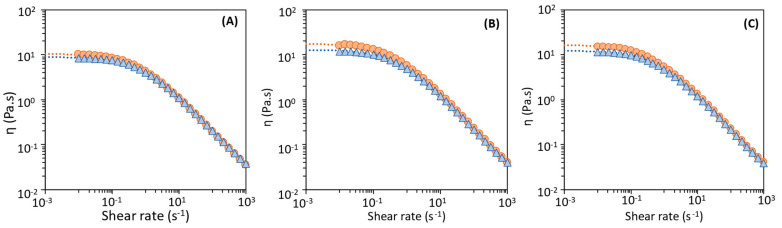
Flow curves of FucoPol aqueous solutions (1.0 wt%): (**A**) Method 1, (**B**) Method 2, (**C**) Method 3; samples F-i_100_ (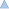) and Fi_30_ (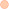); Dotted lines represent the Cross model (*n* = 3).

As shown in Figure 6, the aqueous solutions of all FucoPol samples displayed a shear-thinning fluid behavior, typical of high molecular weight polysaccharides [48] that agrees with previous studies [11,24,27,29]. Nevertheless, slight differences are noticed among the samples, namely, a lower apparent viscosity for the samples obtained with Method 1 (Figure 6A) compared to those of Methods 2 and 3 (Figure 6B,C). Moreover, the samples recovered with the 30 kDa MWCO membrane had a slightly higher viscosity for all methods. These findings can be explained by the lower inorganic salts content of samples F-2_30_ and F3_30_ (Figure 3). Xue et al. [49] reported that the presence of inorganic salts typically decrease the viscosity of polysaccharides (e.g., welan) solutions due to the electrostatic interaction established with the polysaccharide chain.

A non-Newtonian mathematical model, the Cross model, was fitted from the experimental results (Figure 6) with the resulting parameters given on Table 3. Samples F-1_100_ and F-1_30_, recovered from the cultivation broth by Method 1 (diafiltration-ultrafiltration), had the lowest *η*_0_ values (8.89 ± 0.04 and 10.40 ± 0.84 Pa·s, respectively), as well as the lowest τ (1.26 ± 0.07 and 1.58 ± 0.08 s, respectively), thus confirming the lowest apparent viscosity of their aqueous solutions. The highest *η*_0_ values were observed for FucoPol samples F-2_30_ and F-3_30_ (17.40 ± 0.04 Pa·s and 16.30 ± 0.04, respectively), both recovered with the 30 kDa membrane, but with different methods. The “m” constant values are similar for all samples and agree with those reported for FucoPol (0.645 ± 0.024) [24,27]. in close agreement with former data published by Morris [50] for polysaccharides (*m* = 0.76).

**Figure 7:**
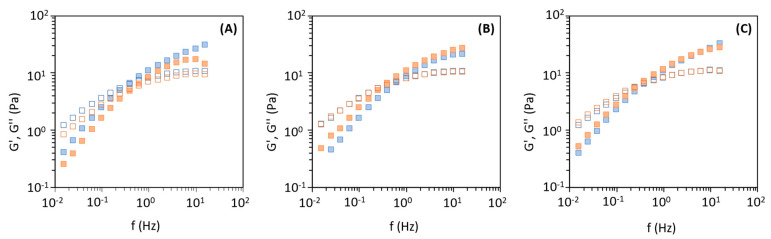
Mechanical spectrum of FucoPol aqueous solutions (1.0 wt%), G′ (closed square) and G′′ (open square): (**A**) Method 1, (**B**) Method 2, (**C**) Method 3; F-i_100_, blue; F-i_30_, orange. Data are shown as the average ± standard deviation (SD) (*n* = 3).

The mechanical spectra (Figure 7) of the six FucoPol samples in aqueous media showed that the loss modulus (G′′) is higher than the storage modulus (G′), indicating a liquidlike behaviour [24,29]. The mechanical spectra for all samples are quite similar, with G′ increasing at a higher rate than G′′ at the given frequency, with the crossover of dynamic moduli being perceived at similar frequencies. Nevertheless, for samples F-1_100_ and F-1_30_, the crossover occurred at a slightly higher frequency (0.6 Hz) (Figure 7A) than for the remaining samples (0.5 Hz for samples F-2_100_ and F-3_100_, and 0.4 Hz for samples F-2_30_ and F-3_30_) (Figure 7B,C). The higher the viscosity the lowest will be the energy necessary to store energy and observe a Gʹ Gʹʹ crossover, i.e., the crossover will occur at lower frequencies [29]. In previous studies, FucoPol aqueous solutions of similar concentration (1.0–1.2 wt%) were reported to have dynamic crossover values occurring at higher frequencies (2.8–10 Hz) [24,27,29,51]. Such discrepancies might be related to the extraction method used in previous studies (dialysis with 10–12 kDa membranes) [23,27].

### 3.4. Emulsion Forming and Stabilizing Capacity

**Figure 8:**
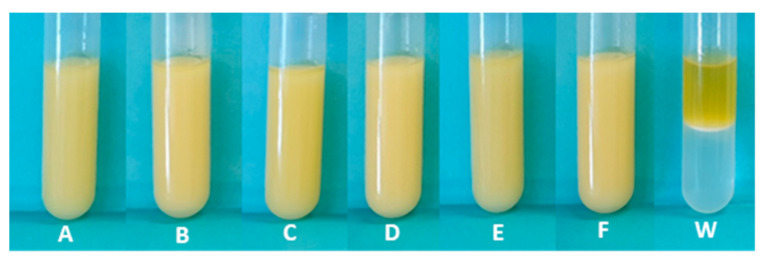
Olive oil/ FucoPol (1.0 wt%) emulsions (2:3 O/W ratio) after 24 h for the extracted samples. Method 1 (F-1_100_, (**A**); F-1_30_, (**B**)); Method 2 (F-2_100_, (**C**); F-2_30_, (**D**)); Method 3 (F-3_100_, (**E**); F-3_30_, (**F**)); (**W**)-blank sample, oil/water.

The ability of FucoPol to form and stabilize emulsions was assayed for all extracted samples by preparing emulsions using olive oil as the test hydrophobic compound. The assays consisted in mixing each FucoPol sample, at a concentration of 1.0 wt%, with olive oil, at a 2:3 (*v/v*) ratio (Figure 8). The results show that all samples efficiently emulsified olive oil (E24 = 98%) (Table 4). According to Willumsen and Karlson [52], a good emulsifier has E24 values equal to or above 50%. FucoPol’s ability to form and stabilize emulsions with different hydrophobic compounds (e.g., cedarwood oil, sunflower oil, corn oil and, rice bran oil), at different O:W ratios (e.g., 1:4, 2:3, 3:2, 4:1), was previously demonstrated with reported E24 values ranging from 41 to 80% [23,27,53].

**Figure 9:**
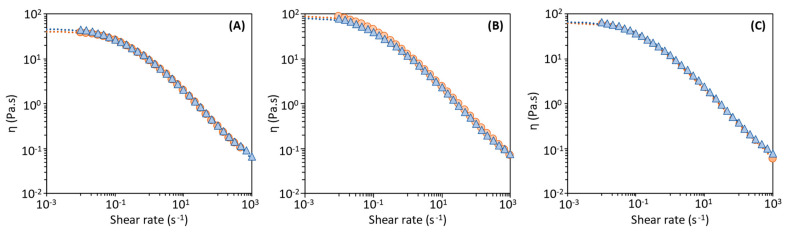
Flow curves for the prepared olive oil/FucoPol (1.0 wt%) emulsions (2:3 O/W ratio): (**A**) Method 1, (**B**) Method 2, (**C**) Method 3; samples F-i_100_ (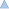) and Fi_30_ (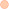). Dotted lines represent the Cross model. (*n* = 3).

As shown in Figure 9, the olive oil/FucoPol emulsions exhibited shear-thinning flow behaviour. Moreover, the *η*_0_ of the emulsions was considerably higher (Table 4) compared to the corresponding aqueous solutions (Table 3). As reported by Calero et al. [54], the increase of the apparent viscosity of the resulting emulsions relatively to the polymer aqueous solutions is typically explained by an increase of polymer chain entanglements within the aqueous layer surrounding the oil droplets, leading to a higher resistance to flow under steady shear. The lowest *η*_0_ was observed for the emulsions prepared with samples F-1_100_ (46.5 ± 5.3 Pa·s) and F-1_30_ (41.3 ± 19.1 Pa·s), which were also those displaying the lowest viscosity in aqueous solution (8.89 ± 0.62 and 10.40 ± 0.84 Pa·s, respectively). The highest *η*_0_ value was noticed for the emulsion prepared with sample F-2_30_ whose aqueous solutions also displayed the highest *η*_0_ (17.40 ± 0.04 Pa·s) (Table 3).

## 4. Discussion

The results obtained in this study show that Method 2 (ultrafiltration with the 30 kDa membrane—sample F-2_30_) reached the best performance in terms of operation time and water consumption, together with good FucoPol recovery (Table 5). Compared to Method 1 (diafiltration-ultrafiltration with a 100 kDa membrane—sample F-1_100_) that was used in previous studies [13,28,33], there was a reduction of the extraction time from 105 ± 6 min to 66 ± 6 min and a reduction of the water consumption by 55%. This is translated in terms of energy, water and time savings for the overall FucoPol production process that are of special relevance for the process scale up. Thus, the optimized downstream procedure contributes to render the process more sustainable from an economic and environmental point of view.

On the other hand, the optimized procedure also yielded FucoPol with a higher purity degree, as shown by its lower protein and inorganic salts that were reduced by 12 and 53%, respectively (Table 5), compared to the previously used procedure (Method 1). Of special relevance is the fact that FucoPol molecular structure was not apparently affected by the conditions, as shown by its similar sugar and acyl groups composition, identical FT-IR spectrum and similar molecular mass distribution. There was also no significant impact on FucoPol’s thermal degradation profile, despite the slightly lower adsorbed water content and char yield.

Interestingly, the aqueous solutions prepared with FucoPol recovered by the optimized method displayed a higher apparent viscosity (Table 5). The ultrafiltration process may originate shearing stress which can enhance dissociation of polysaccharide aggregates, increasing the fraction of polymer chains truly dissolved in solution, resulting in viscosity augmentation [11,50]. For Methods 2 and 3, the module was operated solely/mostly in the ultrafiltration mode. This difference could explain the highest viscosity of samples F-2_i_ and F-3_i_ aqueous solutions compared to those recovered by Method 1. The highest TMP experienced in Method 2 (0.73 ± 0.05 and 0.67 ± 0.20 bar) and Method 3 (0.76 ± 0.11 and 0.71 ± 0.14 bar) compared to Method 1 (0.63 ± 0.13 and 0.62 ± 0.16 bar) (Table 1) might have promoted shearing stress experienced by the polysaccharide chains, thus contributing to their disentanglement. The ability of FucoPol to form emulsion with olive oil was not affected, with the emulsions prepared with the sample recovered with the optimized procedure displaying the same E24 value (98%). Moreover, the resulting emulsions were characterized by a significantly higher apparent viscosity, a feature of relevance for FucoPol’s utilization in cosmetic, pharmaceutical or food products [1,2,5,8,9,55,56,57].

## 5. Conclusions

This study reports the optimized extraction procedure for recovery of FucoPol from the cultivation broth of *Enterobacter* A47, using an ultrafiltration method with a 30 kDa membrane in contrast to a diafiltration-ultrafiltration procedure with a 100 kDa membrane made from the same material chemistry. Compared to methods reported earlier, the optimized downstream procedure allowed water and time savings, concomitant with improved FucoPol recovery and purity. There was no significant impact on the biopolymer’s physical-chemical performances, still improving the rheological properties of the purified biopolymer. The olive oil emulsions stabilized with the extracted FucoPol also disclosed higher viscosity, a characteristic of relevance for FucoPol’s development into pharmaceutical and cosmetic applications.

These findings are also relevant for process implementation at large scale, taking into account the benefits in terms of energy, water and time saving, all factors contributing to making FucoPol production more cost-effective and environmentally sustainable. Additionally, the developed downstream procedure might be applied for the extraction of other EPS.

## Figures and Tables

**Table 1:** Recovery of FucoPol from the cell-free supernatant using Method 1 (diafiltration-ultrafiltration), Method 2 (ultrafiltration) and Method 3 (ultrafiltration-diafiltration-ultrafiltration) with 100 or 30 kDa MWCO membranes. Data are shown as the average ± standard deviation (SD) (*n* = 3).

Method	Membrane (kDa)	Sample	Average TMP (bar)	Extraction Time (min)	Water Consumption (L)	Polymer Recovery (g)
1	100	F-1_100_	0.63 ± 0.13	105 ± 6	9.6 ± 0.0	1.20 ± 0.11
	30	F-1_30_	0.62 ± 0.16	130 ± 6	9.6 ± 0.0	1.38 ± 0.04
2	100	F-2_100_	0.73 ± 0.05	77 ± 8	4.3 ± 0.0	1.22 ± 0.04
	30	F-2_30_	0.67 ± 0.20	66 ± 6	4.3 ± 0.0	1.31 ± 0.05
3	100	F-3_100_	0.76 ± 0.11	81 ± 4	6.0 ± 0.0	1.06 ± 0.09
	30	F-3_30_	0.71 ± 0.14	85 ± 1	6.0 ± 0.0	1.50 ± 0.04

**Table 2:** Physical-chemical characterization of FucoPol samples (Fuc: fucose; Gal: galactose; Glc: glucose; GlcA: glucuronic acid; Pyr: pyruvyl; Succ: succinyl; Acet: acetyl; Mw: molecular weight; PDI: polydispersity index).

Sample	Sugar Monomers (mol%)				Acyl Groups (wt%)			Mw (x106)	PDI	Tdeg (°C)
	Fuc	Gal	Glc	GlcA	Pyr	Succ	Acet	(Da)		
FucoPol (*)	32–36	25–26	28–34	9–10	3.7–14.0	0.6–3.0	3.5–6.8	1.7–5.8	1.3–1.9	268
F-1_100_	38	24	28	7	5.4	1.1	5.4	1.6	1.36	262
F-1_30_	40	24	29	6	5.2	1.0	4.4	1.7	1.74	261
F-2_100_	40	24	29	7	5.2	1.0	5.4	1.4	1.51	261
F-2_30_	40	24	29	7	5.2	1.1	4.6	1.6	1.48	262
F-3_100_	41	24	27	7	5.1	1.0	4.2	1.7	1.70	263
F-3_30_	40	24	29	7	5.3	1.1	5.3	2.0	1.71	262

(*) [13,21,23,25,27,30,31,35].

**Table 3:** Cross model parameters estimated for FucoPol samples (1.0 wt%, *w/v*) recovered from the cultivation broth by the different tested different methods. *η*_0_—apparent viscosity of the second Newtonian plateau (Pa·s); *τ*— relaxation time (s); *m*—dimensionless constant. Data are shown as the average ± standard deviation (SD) (*n* = 3).

Method	Membrane Cut-Off (kDa)	Sample	Cross Model		
			*η*0 (Pa·s)	*τ* (s)	*m*
1	100	F-1_100_	8.89 ± 0.62	1.26 ± 0.07	0.78 ± 0.00
	30	F-1_30_	10.40 ± 0.84	1.58 ± 0.08	0.78 ± 0.00
2	100	F-2_100_	12.80 ± 0.58	1.89 ± 0.11	0.77 ± 0.00
	30	F-2_30_	17.40 ± 0.04	1.68 ± 0.21	0.78 ± 0.00
3	100	F-3_100_	12.30 ± 1.16	1.77 ± 0.03	0.78 ± 0.00
	30	F-3_30_	16.30 ± 0.04	2.23 ± 0.03	0.78 ± 0.00

RE = $\overset{n}{\underset{i=1}{\sum }}\left(\left|\mathrm{xexp},i\;-\;\mathrm{xcalc},i\right|/\mathrm{xexp}\right)/n\;$is between 0.011 and 0.019.

**Table 4:** Emulsification index (E24) and zero shear viscosity (*η*_0_) for the emulsions prepared with the extracted FucoPol samples and olive oil, at an oil/water (O/W) ratio of 2:3. Data are shown as the average ± standard deviation (SD) (*n* = 3).

Method	Membrane Cut-Off (kDa)	Sample	E_24_ (%)	*η*_0_ (Pa·s)
1	100	F-1_100_	98 ± 0	46.5 ± 5.3
	30	F-1_30_	98 ± 0	41.3 ± 19.1
2	100	F-2_100_	98 ± 0	81.5 ± 10.5
	30	F-2_30_	98 ± 0	90.2 ± 4.4
3	100	F-3_100_	98 ± 0	66.9 ± 3.8
	30	F-3_30_	98 ± 0	63.9 ± 6.3

**Table 5:** Overall performance of the developed optimized procedure comprising ultrafiltration with a 30 kDa membrane, comparing to the previously used method (diafiltration-ultrafiltration with a 100 kDa membrane).

Parameter	Ultrafiltration
	30 kDa Membrane
Process	
Water consumption	↓ 55%
Extraction time	↓ 37%
Product recovery	↑ 10%
FucoPol composition	Similar (↑ fucose content)
Contaminants	
Protein content	↓ 12%
Inorganic salts content	↓ 53%
Molecular mass distribution	
Mw	Unchanged
PDI	Similar
Thermal properties	Unchanged
Apparent viscosity	↑ 96%
Emulsion forming capacity	
E24	Unchanged (98%)
Emulsion viscosity	↑ 100%

## Data Availability

Data will be available upon request.
